# Ca_v_1.2, Cell Proliferation, and New Target in Atherosclerosis

**DOI:** 10.1155/2013/463527

**Published:** 2013-05-12

**Authors:** Nikolai M. Soldatov

**Affiliations:** Humgenex, Inc., Kensington, MD 20895, USA

## Abstract

Ca_v_1.2 calcium channels are the principal proteins involved in electrical, mechanical, and/or signaling functions of the cell. Ca_v_1.2 couples membrane depolarization to the transient increase in intracellular Ca^2+^ concentration that is a trigger for muscle contraction and CREB-dependent transcriptional activation. The CACNA1C gene coding for the Ca_v_1.2 pore-forming *α*_1C_ subunit is subject to extensive alternative splicing. This review is the first attempt to follow the association between cell proliferation, Ca_v_1.2 expression and splice variation, and atherosclerosis. Based on insights into the association between the atherosclerosis-induced molecular remodeling of Ca_v_1.2, proliferation of vascular smooth muscle cells, and CREB-dependent transcriptional signaling, this review will give a perspective outlook for the use of the CACNA1C exon skipping as a new potential gene therapy approach to atherosclerosis.

## 1. Introduction

It has been long known that Ca_v_1.2 calcium channel blockers inhibit human brain tumor [1], pancreatic cancer [2, 3], breast cancers [4] and small cell lung cancer [5] because they inhibit cell proliferation and DNA synthesis. Correlation between the oncogenic transformation and expression of both Ca_v_1.2 and Ca_v_3 channels was demonstrated in spontaneously immortalized 3T3 fibroblasts [6, 7] suggesting that both types of calcium channels may play a role in cell proliferation. Indeed, studies showed that Ca_v_3 (T-type) calcium channels regulate proliferation, for example, of BC_3_H1 cells [8], vascular smooth muscle (VSM) cells [9], and glioma, neuroblastoma, and neuroblastoma × glioma hybrid cells [10]. Unlike the majority of cells, including the listed ones, normal human fibroblasts express only Ca_v_1.2 [11]. The pore-forming *α*_1C_ subunit of this channel was cloned from human fibroblasts and identified as a “short” (exon 1) isoform of the *α*_1C_-coding gene CACNA1C [12]. A variety of Ca_v_1.2 calcium channel blockers, including dihydropyridines (DHPs) nifedipine and nicardipine, as well as diltiazem and verapamil inhibit cell proliferation and DNA synthesis in fibroblasts [13]. Thus, human fibroblast is an excellent cell type to study the roles of Ca_v_1.2 in proliferation not complicated by expression of other Ca_v_ genetic variants, cell transformation, and/or differentiation.

## 2. Ca_**v**_1.2 and Proliferation of Normal Human Fibroblasts

**Figure 1:**
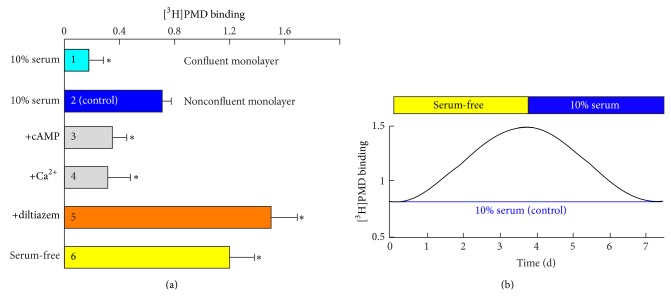
Effect of serum and second messengers on the density of DHP receptors in normal human fibroblasts. (a) On day 2 after the plating of fibroblasts at high (bar 1) or low density (bars 2–6), the growth medium was supplemented with 1 mM 8Br-cAMP (bar 3), 1 *μ*M Ca^2+^ ionophore A23187 (bar 4), 1 *μ*M D-cis-diltiazem (bar 5), or growth medium was replaced by serum-free Eagle's medium containing 0.1% BSA (bar 6). After 4 days, the density of DHP receptors was measured at 2 nM [^3^H]-PMD as pmol [^3^H]PMD specifically bound per 10^6^ cells of confluent (bar 1, >2 × 10^4^ cells/cm^2^) or nonconfluent monolayers (bars 2–6, 3–5 × 10^3^ cells/cm^2^). Mean of 5-6 measurements ± SEM. ^*^ *P* < 0.05. (b) Effect of serum deprivation on the density of DHP receptors in fibroblasts. On day 2 after plating, sparse cultures of fibroblasts were incubated for up to 4 days in serum-free Eagle's medium supplemented with 0.1% BSA. The horizontal bars indicate when serum-free medium was replaced by standard growth medium containing 10% serum. The time course of changes in the density of DHP binding sites was measured as in (a) at different time points (not shown for simplicity) and compared with that in cells not subjected to serum-deprivation (blue line).

Our earlier studies performed on normal human diploid fibroblasts have revealed a number of important features that point to the plasticity of the Ca_v_1.2 expression in response to cell culture conditions, including cell-cell contact inhibition, presence of mitogens, and second messengers. The expression of Ca_v_1.2 in the plasma membrane was measured using the DHP radioligand binding assay. DHPs bind to Ca_v_1.2 with high affinity in equimolar ratio and are excellent probes for the expression of total (functional and dormant) Ca_v_1.2 in the plasma membrane. The test system was based on the measurement of specific binding of 2,6-dimethyl-3-methoxycarbonyl-5-([2,3-^3^H_2_]-n-propoxycarbonyl)-4-(2′-difluoromethoxyphenyl)-1,4-dihydropyridine ([^3^H]PMD, NCBI PubChem CID 14267917) [15] to human embryonic diploid fibroblasts grown in Eagle's medium supplemented with 10% serum. Under standard conditions of incubation (1 h at room temperature in Tris-buffered saline) [^3^H]PMD interacted with *K*_*d*_ ≈ 3.9 nM with a single class of DHP receptors that were present at the maximum density (*B*_max_) of *≈*1.2 pmol/10^6^ cells in a sparse culture of fibroblasts (*≈*3–5 × 1,000/cm^2^) [16]. The turnover rate of DHP receptors is approximately exponential with a half-life of *≈*12 h, as it was estimated from the rate of loss of [^3^H]PMD binding sites in response to the net inhibition of protein synthesis by cycloheximide. With progression to confluent monolayers, the *K*_*d*_ value for [^3^H]PMD binding did not change, but the *B*_max_ value decreased *≈*4 fold (Figure 1(a), compare bars 1 and 2), suggesting that expression of Ca_v_1.2 is responsive to the arrest of fibroblasts proliferation by cell-cell contact inhibition. 

The involvement of Ca_v_1.2 in proliferation of human fibroblasts was further supported by the finding that concentration of DHP receptors, and, respectively, of Ca_v_1.2 is strongly affected by mitogens and second messengers. Demonstrating a remarkable plasticity of the Ca_v_1.2 expression in normal human fibroblasts (Figure 1(b)), serum deprivation induced a 2-fold increase in the density of DHP receptors that reached its maximum after 3-4 days of cultivation in the absence of serum (compare also bars 2 and 6 in Figure 1(a)). The elevation of the Ca_v_1.2 expression was fully reversible and highly sensitive to serum and other mitogens. An addition of 10% serum reduced the density of DHP receptors to the initial level with almost the same time course (Figure 1(b)). Thus, inhibition of cell growth, proliferation, and DNA synthesis in fibroblasts by serum deprivation stimulates expression of Ca_v_1.2 with a time course comparable with the DHP receptor turnover rate. 

The response of fibroblasts to serum deprivation by boosting Ca_v_1.2 expression is essentially identical to that caused by the inhibition of Ca_v_1.2 by 1 *μ*M diltiazem (Figure 1(a), compare bars 5 and 6), the calcium channel blocker that does not compete with DHPs for binding with the channel. In fact, diltiazem was present in the DHP binding assay medium throughout all experiments to enhance the affinity of the DHP probe to the channel receptor [17]. Thus, it is reasonable to suggest that by boosting the Ca_v_1.2 expression, the cell recruits more routes for the Ca^2+^ entry through the plasma membrane to overcome the lack of mitogens in serum deprivation or lack of conducting channels in the presence of diltiazem, both aimed at supporting cell proliferation until cell-cell contact inhibition terminates it and turns the cells into a quiescent state.

If this hypothesis is true, then the addition of DHP-insensitive routes for Ca^2+^ entry through the plasma membrane should eliminate the need in higher level of Ca_v_1.2 expression. This observation exactly has been made when the serum-stimulated cells were supplemented with Ca^2+^ ionophore A23187 (Figure 1(a), bar 4). The A23187-induced Ca^2+^ entry dramatically reduced the cellular expression of Ca_v_1.2. A similar effect was observed with 8Br-cAMP, the plasma membrane-permeable derivative of cAMP, showing that stimulation of the alternative cAMP-dependent cell signaling pathway may reduce the needs in Ca_v_1.2 in proliferation of fibroblasts.

**Figure 2:**
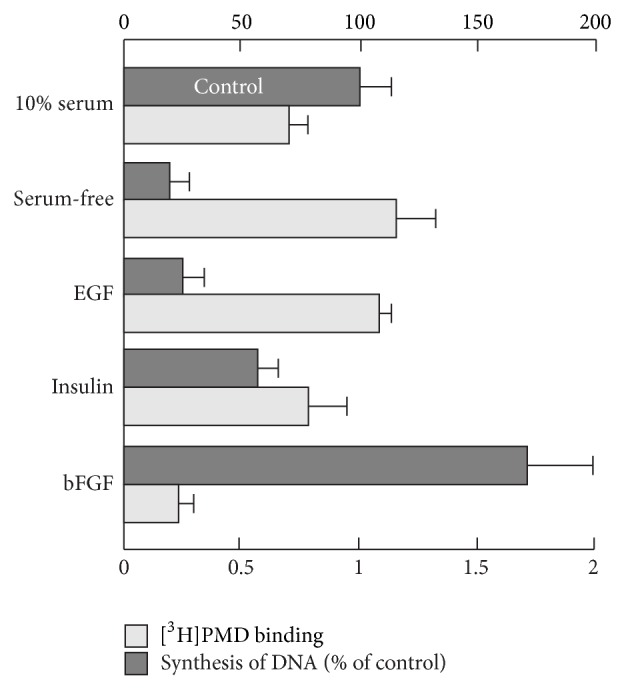
Effect of mitogens on the density of DHP receptors (open bars) and cell proliferation (dark bars) in normal human fibroblasts. On day 2 after cell plating, the growth medium was replaced for 4 days with serum-free Eagle's medium supplemented with 0.1% BSA. Cells were incubated for 4 days in the presence of 10% serum (control), 0.1% BSA (serum-free), epidermal growth factor (EGF, 15 ng/mL), insulin (10 *μ*g/mL), or basic fibroblast growth factor (bFGF, 2 ng/mL). The density of DHP receptors was measured at 2 nM [^3^H]-PMD as pmol [^3^H]-PMD specifically bound per 10^6^ cells of nonconfluent monolayers (3–5 × 10^3^ cells/cm^2^). Cell proliferation was estimated from the synthesis of DNA measured by [^14^C]thymidine incorporation.

Stimulation of Ca_v_1.2 expression in fibroblasts, arrested in the quiescent state by serum deprivation, strongly depends on cell proliferation. The measurement of [^14^C]thymidine incorporation as an assay for DNA synthesis in cells showed (Figure 2, dark bars) that individual mitogens stimulated cell proliferation in the order bFGF > insulin > EGF. In contrast, in that very order the same mitogens suppressed expression of Ca_v_1.2 in fibroblasts as evidenced by the measurement of DHP receptor binding (open bars). Consistent with relatively low cell toxicity of dihydropyridines, blockade of Ca_v_1.2 attenuated entry of cells into the S phase of cell cycle [18]. Thus, the plasticity of Ca_v_1.2 in fibroblast proliferation is associated with transient Ca_v_1.2 expression responsive to both primary effectors and second messengers of cell proliferation. 

## 3. Ca_**v**_1.2 Variability

One of the most important features of Ca_v_1.2 is its remarkable molecular diversity. The channel consists of the pore-forming *α*_1C_ protein, which binds calcium channel blockers. Three types of regulatory subunits, *β*, *α*_2_ *δ*, and calmodulin (CaM), are constitutively tethered to *α*_1C_. All these subunits are products of different genes that are located on different chromosomes. Ca_v_1.2 shares the accessory subunits with other Ca_v_1 and Ca_v_2 channels. Moreover, *α*_1C_, *β*, and *α*_2_ *δ* are subject to individual alternative splicing that generates a large diversity of Ca_v_1.2 channel complexes. The functional significance of the Ca_v_1.2 splice variation is poorly understood not only because of the difficulties in identification of individual splice variants in the naturally occurring oligomeric proteins. The tendency of Ca_v_1.2 to form large clusters in the plasma membrane [19–23] as well as homo- and heterooligomerization of *β* subunits [24] create additional major challenges for the investigation of Ca_v_1.2 in regulation of cell proliferation, differentiation, and other functions—not to speak of an adequate elucidation of their roles in disease and search for new therapeutic approaches.

**Figure 3:**
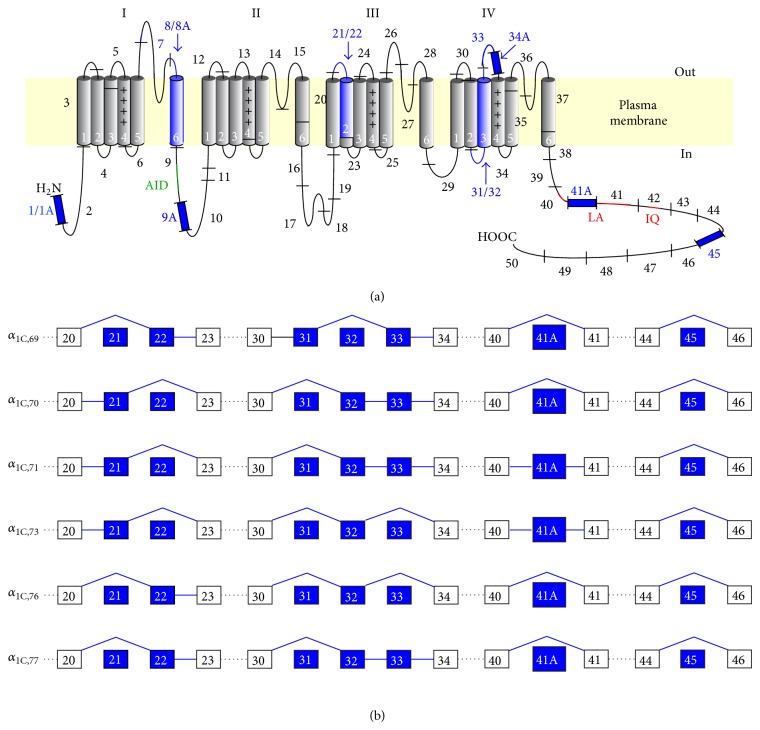
Molecular diversity of Ca_v_1.2. (a) Transmembrane topology of the Ca_v_1.2 *α*_1C_ subunit in schematic diagram. Four regions of homology (I–IV), each composed of 6 transmembrane segments (numbered), are folded around the central pore. The polypeptide sequence is segmented by black bars and sequentially numbered (1–50) according to the CACNA1C genomic map [14]. The segments corresponding to the invariant (black/gray) and alternative (blue) exons are outlined. *α*-interaction domain (AID) of a constitutive *β*-binding is shown in green. In the CaM-binding domain (CBD), apo-CaM and Ca^2+^-CaM are shared between LA and IQ motifs (red), respectively. (b) Alternative splicing of human fibroblast CACNA1C in the region of exons 20–46. The following splice variants were studied: *α*_1C,69_ (GenBank #z34809, splice variant lacking alternative exons (Δ) 21, 32, 41A, and 45), *α*_1C,70_ (z34810, Δ 22, 31, 41A, and 45), *α*_1C,71_ (z34811, Δ 22, 31, and 45), *α*_1C,73_ (z34812, Δ 22, 31, 33, and 45), *α*_1C,76_ (z34814, Δ 21, 31, 33, 41A, and 45), and *α*_1C,77_ (z34815, Δ 21, 31, 41A, and 45).

Molecular cloning showed that *α*_1C_ transcripts in human fibroblasts are composed of exons 1–50 [14] with alternative splicing of exons 21/22 and 31/32 and constitutive splicing of exons 33 and 45 (Figure 3(a)) [12]. However, variability associated with exons 1, 7, 8, 9, 34, 41, and 42 that were later found in human hippocampus, heart, and VSM cells [25–30] was not observed in fibroblasts. 

It is not known which Ca_v_1.2 splice variants are expressed in fibroblasts in response to serum deprivation, and whether they are structurally different from those present in proliferating cells. However, electrophysiological properties of the three of fibroblast *α*_1C_ splice variants (coexpressed with *β*_1a_ and *α*_2_ *δ*-1) were compared in *Xenopus* oocyte expression system [31]. Characteristics of the voltage-dependence and kinetics of inactivation of the barium current through the *α*_1C,70_ channel encoded by the CACNA1C transcript lacking exons 22, 31, 41A and 45 were found to be very similar to those recorded with *α*_1C,77_ (lacking exons 21, 31, 41A and 45) and *α*_1C,76_ (also lacking exon 33) (Figure 3(b)). However, voltage dependence of the DHP inhibition of the current was significantly different in the *α*_1C,70_ and *α*_1C,77_ channels: the IC_50_ values for the concentration dependence of the barium current inhibition by (+)isradipine, almost identical (6.2 and 7.3 nM, resp.) at −40 mV, were significantly different at −90 mV (680 and 79 nM for *α*_1C,70_ and *α*_1C,77_, resp.). While such a difference in the pharmacological properties of the exon 21- and exon 22-splice variants is deemed unimportant in the case of fibroblasts, it may significantly contribute to the tissue specificity of this major class of calcium channel blockers in cardiac, vascular, and other responsive cells affected by cardiovascular diseases. 

## 4. Ca_**v**_1.2 in Atherosclerosis

Atherosclerosis is perhaps the single most deadly disease, leading to about 600,000 deaths annually in the USA, most of these due to the progression of the disease to heart attack or stroke [32]. In spite of significant efforts, the molecular mechanisms of atherosclerosis are not currently well understood, and effective molecular targets for prevention and treatment are not elaborated. Atherosclerosis is an inflammatory process in medium and large size arteries that causes endothelial perturbation and local release of cytokines, as well as dedifferentiation, proliferation, and migration of VSM cells [33]. Arterial VSM cells constitute the media of the artery and play a crucial role in its elasticity and contractility. Migration of VSM cells from the media to the intima of the arterial wall and proliferation of intimal smooth muscle cells are the major early events in the formation of atherosclerotic lesions. Recent advances in molecular genetics studies have revealed that genetic polymorphisms significantly influence susceptibility to atherosclerotic vascular diseases [34]. However, none of the discovered susceptibility genes was directly implicated for proliferation and migration of VSM cells, one of the major pathophysiological responses to atherosclerosis at the cellular level.

 The presence and activity of Ca_v_1.2 calcium channels in VSM cells has been established both in patch clamp and molecular cloning experiments [29, 35–38]. Ca_v_1.2 calcium channels play a major role in atherosclerosis because they are essential for Ca^2+^ signal transduction in VSM cells. Contraction of VSM cells is triggered by the Ca^2+^ current (*I*_Ca_) through Ca_v_1.2, and thus is affected by Ca^2+^ channel blockers. Since the 1990s, it is known that DHPs, particularly, the charged 2-aminoethoxymethyl DHP derivative amlodipine [39], exert a number of vasoprotective effects, including potent antiatherogenic action [40], inhibition of migration of VSM cells [41], and reduction of arterial intimal-medial thickening and plaque formation [42, 43]. Although it is tempting to link this activity in part to pleiotropic effects of calcium channel blockers [44], it is the inhibition of proliferation that essentially underlies it [45]. The density of DHP receptors was found to depend on VSM cell proliferation [46]. Association of Ca_v_1.2 with mitogenesis in VSM cells is supported by the findings that DHPs reduced DNA synthesis stimulated by serum and PdGF [47–49], serotonin [50], EGF [51], and H_2_O_2_ (in mesangial cells) [52]. Expression of Ca_v_1.2 in VSM cells was shown to be cell cycle dependent, with the highest calcium current density in G_1_ phase [53]. Whether these changes are reflected in the molecular repertoire of the Ca_v_1.2 splice variants is the issue particularly important for the elucidation of new therapeutic targets in diseases leading to pathogenic proliferation of VSM cells, such as atherosclerosis. 

**Figure 4:**
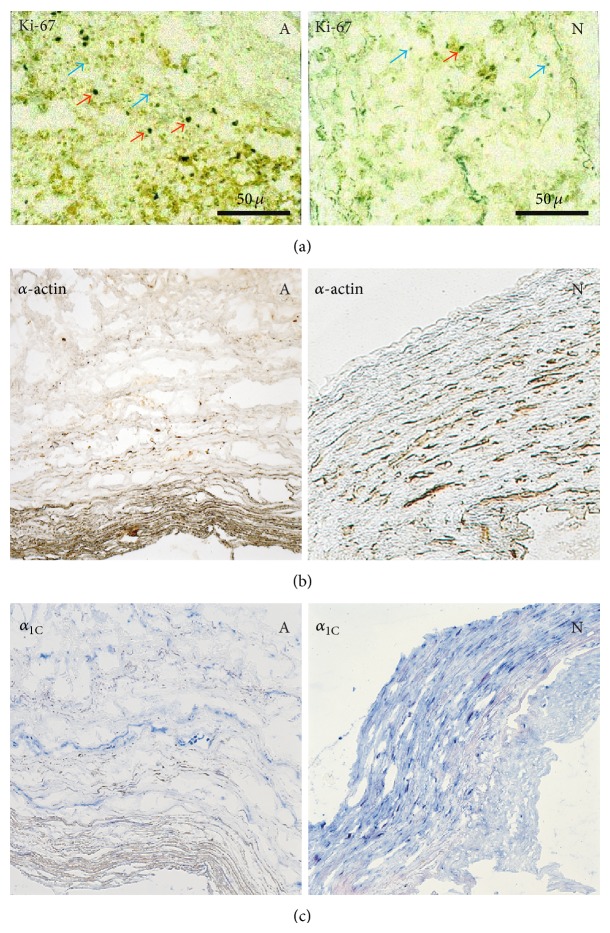
Reduced expression of *α*_1C_ in atherosclerotic (A) region of aorta as compared to the adjacent nonaffected (N) region. From (a) to (c): detection of proliferating cells in an exemplar biopsy of an artery by an antibody against human nuclear protein Ki-67 (a). Red arrows highlight some proliferating nuclei (black staining), while blue arrows exemplify nonproliferating nuclei (light grey). Staining with antibodies against smooth muscle *α*-actin (b) and against *α*_1C_ (c). Scale bars, 50 *μ*m.

 Investigation of the *α*_1C_ alternative splicing in VSM cells [28, 54, 55] revealed the involvement of a number of CACNA1C exons generating impressive diversity of human vascular *α*_1C_ that includes possibly the VSM-specific splicing of exons 9/9a [35, 55] and exon 34 [28]. To establish an association between the disease and CACNA1C splice variation at the level of cell, we have completed [26] the single-gene profiling of the *α*_1C_ molecular remodeling in VSM cells of an artery caused by atherosclerosis. The VSM cells were identified in frozen sections of six surgical biopsy samples of femoral and carotid arteries by immunostaining with an antibody against smooth muscle *α*-actin [56], used as a marker for VSM cells. The *α*-actin staining correlated with immunostaining by an anti-*α*_1C_ antibody in serial sections and was reduced in atherosclerotic regions (Figure 4) consistent with dedifferentiation of VSM cells [57, 58]. The reduced expression of *α*_1C_ at the protein level was corroborated by the quantitative RT-PCR data showing that the relative *α*_1C_ mRNA level in VSM cells (normalized to 18S RNA) was reduced 3.7 ± 0.9 fold (mean ± SEM) in the atherosclerotic region. Overall, the reduced expression of *α*_1C_ caused by the locally elaborated cytokines in the atherosclerotic regions of arteries resembles the reduced expression of DHP receptors observed in fibroblasts exposed to mitogens and/or second messengers after serum deprivation (Figure 1). 

 To find out whether the altered expression of *α*_1C_ in atherosclerotic VSM cells is accompanied by changes in the CACNA1C alternative splicing pattern, we isolated the immunohistochemically identified VSM cells by laser-capture microdissection from adjacent regions of arteries affected and not affected by atherosclerosis and identified the CACNA1C splice variants by RT-PCR. Our findings revealed an extended repertoire of the exon 21 *α*_1C_ splice isoforms in nonatherosclerotic VSM cells characterized by a complex splicing pattern of exons 9, 9A, 31–34, and 41A, including the electrophysiologically characterized *α*_1C,71_ (GenBank # z34811), *α*_1C,73_ (z34812), *α*_1C,125_ (AY830711), *α*_1C,126_ (AY830713), and *α*_1C,127_ (AY830712) splice isoforms. However, only the exon 22 isoform of *α*_1C_  (*α*_1C,77_) was identified in atherosclerotic VSM cells. Thus, the switch of the CACNA1C alterative splicing from exon 21 to exon 22 (Figure 3(b)) is a molecular signature of the Ca_v_1.2 remodeling of VSM cells to a pathophysiological proliferating state in atherosclerosis. The age, gender, ethnicity, drug exposure, and other co-morbid conditions did not appreciably affect this common pattern of the *α*_1C_ splice variation in VSM cells in response to atherosclerosis.

**Figure 5:**
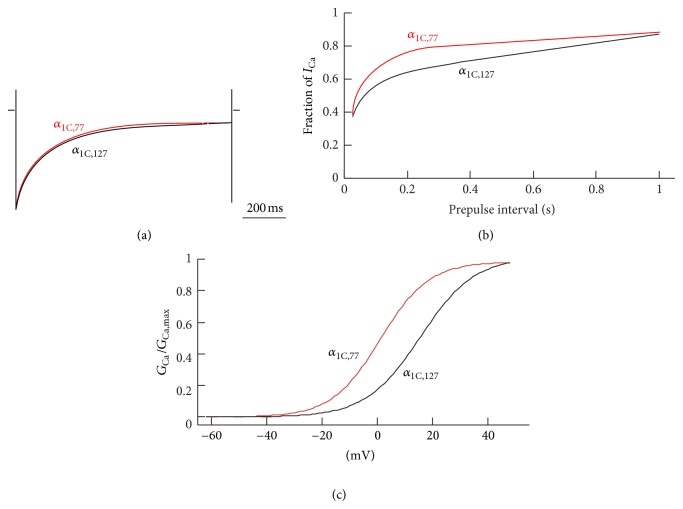
Comparison of electrophysiological properties of *I*_Ca_ through the “atherosclerotic” *α*_1C,77_ and “normal” *α*_1C,127_ channels expressed in *Xenopus* oocytes with *α*_2_ *δ*-1 subunit and the primary cardiac *β*_2a_ subunit and measured with 2.5 mM Ca^2+^ as the charge carrier. (a) Representative traces of *I*_Ca_ evoked by 1 s step depolarizations to +20 mV from *V*_*h*_ = −90 mV and normalized to the same amplitude. (b) Fractional recovery of *I*_Ca_ from inactivation. (c) Averaged activation (*G*/*G*_max_-*V*) curves fit by the Boltzmann function. A 1 s test pulses in the range of −40 to +50 mV (10-mV increments) were applied from *V*_*h*_ = −90 mV with 30-s intervals.

 Careful electrophysiological analysis exhibited a number of differences in the properties of the “atherosclerotic” *α*_1C,77_ channel as compared to the *α*_1C_ isoforms in healthy VSM cells. The largest differences were found between the *α*_1C,77_ and *α*_1C,127_ channels (Figure 5). In response to step depolarization applied from the holding potential of −90 mV, both channels generate calcium currents (*I*_Ca_) that inactivate with almost identical kinetics (Figure 5(a)). However, we found that *I*_Ca_ through the *α*_1C,77_ channel recovers from inactivation significantly faster than that in *α*_1C,127_ (Figure 5(b)) and other *α*_1C_ isoforms present in healthy VSM cells. This finding suggests that alternative splicing in atherosclerosis may affect vascular tone as a result of the increase in the *I*_Ca_ density in VSM cells. A hyperpolarization shift of the activation curve for the atherosclerotic *α*_1C,77_ channel variant, as compared to Ca_v_1.2 in healthy VSM cells (Figure 5(c)), may also result in an increase of calcium entry in VSM cells. However, the overall 3-4-fold reduction in the expression of Ca_v_1.2 in atherosclerotic VSM cells may scale down some of the observed electrophysiological changes. 

## 5. CACNA1C Exon 22 as a New Therapeutic Target in Atherosclerosis

**Figure 6:**
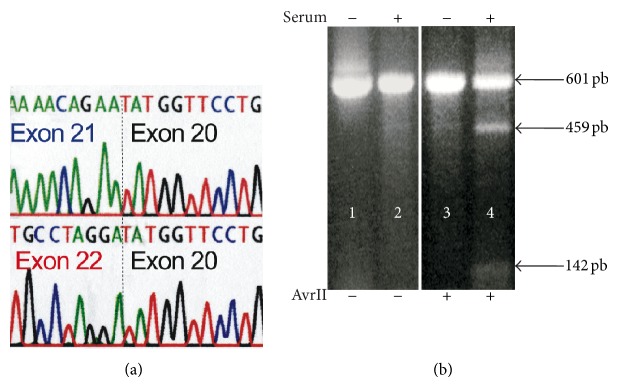
(a) Trace diagrams of DNA sequencing of the *α*_1C_ PCR amplification products of healthy (top row) and diseased VSM cells (bottom row) using an antisense primer composed of nucleotides 2923–2939 (z34815). A boundary with the sequence of invariant exon 20 is marked by a vertical dotted line. (b) Evidence that the AvrII-sensitive exon 22 isoform of *α*_1C_ is expressed only in proliferating VSM cells. Primary aortic cells were grown to confluent monolayer in 5% serum before serum-deprivation for 5 days (lanes 1 and 2). Then, the cells were replated at low density in 5% serum for 3-4 days (lanes 3 and 4). Total RNA was isolated, and exon 21/22 isoforms were identified by RT-PCR and AvrII restriction analysis (lanes 2 and 4).

Direct DNA sequencing of the crude PCR amplification products indicated that the switch to the exon 22 isoform of vascular *α*_1C_ was almost complete in atherosclerosis because no distortion of the nucleotide peaks in the region of exon 21/22 was seen when compared to the exon 20 invariant region (Figure 6(a)). Thus, Ca_v_1.2 underwent almost quantitative exon 21/22 remodeling in VSM cells of diseased artery regions.

Although the cellular mechanisms leading to the CACNA1C exon 21/22 switch may be very complex, the association with VSM cell proliferation is obvious. Indeed, a similar exon switch was observed in primary human aortic cells in culture after the quiescent nonproliferating cells, containing predominantly exon 21 splice variants, were exposed to serum (Figure 6(b)). Unlike exon 21, exon 22 contains the AvrII restriction site that allows for the assessment of its presence in PCR amplification product of CACNA1C transcripts isolated from the cells. The AvrII-sensitive exon 22 isoform of the *α*_1C_ transcript was not detected in the quiescent nonproliferating aortic cells (Figure 6(b), lane 2). However, when 5% serum was added to the medium with nonconfluent aortic cells, DNA biosynthesis was activated, while the level of the *α*_1C_ transcript decreased *≈*3 fold, and the presence of the AvrII-sensitive exon 22 isoforms of *α*_1C_ was easily detected (Figure 6(b), lane 4). The isoform remodeling simulated in aortic primary cells *in vitro* was not complete as compared to VSM cells in atherosclerotic regions of artery occluded with heavy plaque burden, which were selected for the *α*_1C_ molecular profiling. However, the cell culture results demonstrate that in a different experimental system, there is an obvious association between proliferation of VSM cells, downregulation of the CACNA1C expression, and synthesis of the exon 22 *α*_1C_ isoform. 

 Recent strategies targeting VSM cells to treat cardiovascular diseases suggest indiscriminate disruption of Ca_v_1.2 [59–61]. Is it possible to correct the described CACNA1C splice defects induced by atherosclerosis without affecting the transcripts of the gene lacking the "pathogenic" exon 22? Correction of defective genes responsible for disease development is achieved by gene therapy. Usually it requires an insertion into the genome of a normal gene in place of a defective one causing disease. Such technique, however, is poorly controlled. Currently, one of the most promising, cutting-edge therapeutic approaches to correct defects associated with disease-induced expression of abnormal splice variants is antisense-mediated exon skipping. It is based on the use of antisense oligonucleotides targeting specific exons to be removed. The adenovirus-directed *α*_1C_ exon 22 skipping-induced inhibition of VSM cell proliferation (and, respectively, migration) can be used to rescue VSM cells from remodeling in atherosclerosis. The *α*_1C_ exon 22-skipping will not alter the open reading frame of the *α*_1C_ transcript because alternative exons 21 and 22 are of the equal size (60 nt). The modified nonspliceosomal snRNA U7 gene along with its natural promoter and 3′ elements, exon 22-antisense sequence and supplemented with Sm ribonucleoprotein-binding sequence, may be incorporated into the adenovirus vector for high efficiency transfer [62]. The cytokine receptors (e.g., PdGF-*β* receptor) based recognition targeting of viral liposomes or nanoparticles may be especially advantageous in connection with selective gene transfer to VSM cells affected by atherosclerosis, while reducing the probability of the transfection of other cells.

## 6. Ca_**v**_1.2 and CREB-Dependent Transcriptional Activation

How Ca_v_1.2 activity is translated into a proliferation-effected modality is another important question to be asked. Ca_v_1.2 calcium channels generate a transient rise in cytosolic Ca^2+^-concentration activated by membrane depolarization. Cellular responses associated with the rise of [Ca^2+^]_i_ range from sarcomeric contraction to cell growth and proliferation. Cytoplasmic domains of Ca_v_1.2 have evolved a fairly intricate CaM-dependent signaling mechanism that provides for the negative feedback inhibition of the calcium current, known as Ca^2+^ dependent inactivation (CDI), which is mediated by different determinants of *α*_1C_ [25, 63]. Such a mechanism of CDI, resulting in acceleration of *I*_Ca_ inactivation in response to the rise of intracellular Ca^2+^, was first identified in cardiac Ca_v_1.2 [64]. Similar experiments performed on the recombinant Ca_v_1.2 also showed that the replacement of extracellular Ca^2+^ by Ba^2+^ eliminates CDI, and the channel inactivates by a slower voltage-dependent mechanism [65]. The very fact that two distantly located determinants, one in the pore region responsible for slow inactivation, and the CaM-binding one in the proximal locus of the C-tail, are independently crucial for CDI indicates that not only their specific molecular structure but also their mutual folding and/or interaction are essential. Experimental evidence show that this interaction reacts dynamically to membrane voltage supporting state-dependent transitions of the channel between resting, open, and inactivated conformations, which are essential for CREB-dependent transcriptional activation [66, 67]. 

 CREB is a transcription factor of general importance in a large variety of cells. CREB phosphorylation promotes the activation of genes and is regulated by protein kinases under control of the major second messengers, cAMP and/or Ca^2+^. Indeed, CREB functions as a “molecular determinant of VSM cells fate [68].” CREB content depends on proliferation of VSM cells both *in situ* and in culture. Serum deprivation increased CREB content in VSM cells, while exposure to PdGF decreased it. Consistent with this observation, an overexpression of the constitutively active CREB in VSM cells arrested cell cycle progression. 

**Figure 7:**
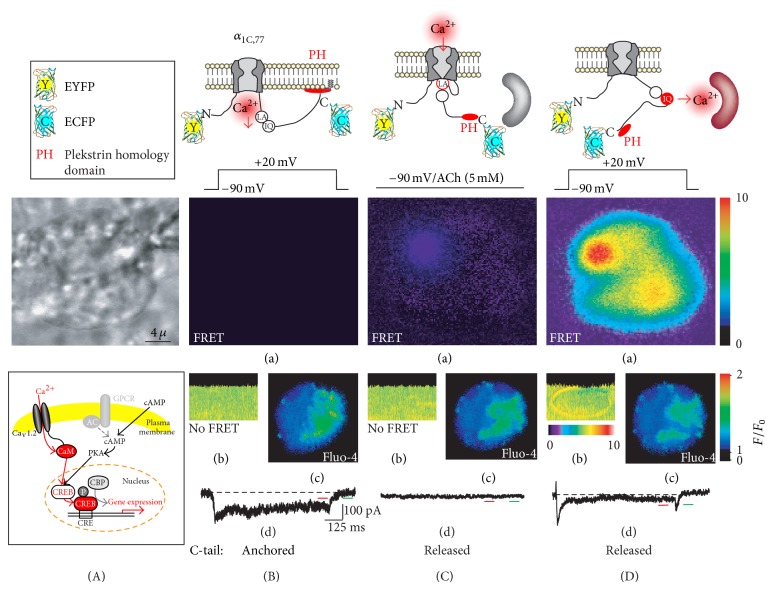
Evidence that Ca^2+^ signal transduction by Ca_v_1.2 to activate CREB-dependent transcription is mediated by the voltage-gated mobility of the *α*_1C_ C-tail carrying the CaM-caged Ca^2+^ and is not directly associated with the increase of [Ca^2+^]_i_. CREB activation was examined under perforated patch conditions in COS1 cells expressing recombinant atherosclerotic *α*_1C,77_ channel. (A) Phase-contrast image of the COS1 cell with a shadow of patch pipette. The cell was expressing the *α*_1C,77_-PH_C_/*β*_1a_/*α*_2_ *δ*-1 channel with the membrane-trapped *α*_1C,77_ subunit C-tail, type 1 muscarinic Ach receptor (to release the anchored C-tail in response to activation by Ach) and EYFP-KID and ECFP-KIX domains, both supplemented with nuclear localization sequences. Inset at the bottom: Ca_v_1.2- and cAMP-dependent cell signaling pathways mediating CREB-dependent transcription. CBP, CREB-binding protein; CRE, cAMP-response element. (B) Plasma-membrane anchoring of the *α*_1C,77_ C-tail (see, schematic diagram) inhibits CREB activation in spite of robust *I*_Ca_ evoked by depolarization. (a) A 100-ms images of FRET between EYFP-KID and ECFP-KIX were recorded at the end of the 12th +20-mV depolarization step applied every 10 s for 1 s from *V*_*h*_ = −90 mV. (b) A corresponding representative image of corrected FRET ratio (in a “3D” MetaMorph transformation) recorded within 50-ms windows at −90 and at the end of the last test pulse of the applied train of pulses. (c) An increase of [Ca^2+^]_i_ detected by Ca^2+^ indicator Fluo4. (d) Representative trace of *I*_Ca_ (20 mM Ca^2+^ in bath medium) evoked by depolarization to +20 mV from *V*_*h*_ = −90 mV showing the sustained component of Ca^2+^ conductance due to the C-tail anchoring. (C) ACh-stimulation of the M1AChR to unbind the *α*_1C_ C-tail caused activation of IP_3_-dependent Ca^2+^ release (panel (c)) but did not show substantial activation of CREB transcription at *V*_*h*_ = −90 mV (panel (a)). (D) CREB-dependent transcription was activated only in response to depolarization applied to membrane channel with the *α*_1C,77_ C-terminal tail released from the plasma membrane to assume functional voltage-dependent conformation.

To investigate the association of Ca_v_1.2 with CREB transcriptional activation at the molecular level, we combined patch clamp with fluorescent resonance energy transfer (FRET) microscopy in the live cell. In voltage clamped cells, FRET provided optical measurements under state-dependent conditions showing that the shorter N-terminal tail of *α*_1C_ (e.g., *α*_1C,77_) does not rearrange vis-à-vis the plasma membrane in response to voltage gating [25]. In sharp contrast, the C-tail shows voltage-dependent conformational rearrangements, which are much larger in size than that, for example, in the potassium K_v_2.1 channel [69]. Measurements of *I*_Ca_ and corrected FRET between the enhanced yellow (EYFP) and cyan fluorescent proteins (ECFP), genetically attached, respectively, to the N- and C-termini of *α*_1C,77_ showed no significant effect on voltage-dependence and kinetics of the channel current. However, there was a substantial increase in FRET signal accompanying inactivation of the channel that was fully reversible upon its transition into the resting state in response to hyperpolarization [66]. The plasma-membrane anchoring of the *α*_1C_ C-tail by the fusion of the pleckstrin homology domain (PH) eliminated CDI but not *I*_Ca_. Do the voltage-gated conformational rearrangements of the *α*_1C_ C-tail, and CDI, play a role in Ca^2+^ signal transduction that is utilized in Ca^2+^-induced activation or CREB-dependent transcription? To answer this question, we used the test system based on the measurement of interaction between KID domain of CREB and KIX domain of coactivator CREB-binding protein (CBP, Figure 7(A), inset) under voltage-clamp conditions by monitoring FRET between EYFP-KID and ECFP-KIX [70]. Ca^2+^-dependent phosphorylation of KID stimulates its binding to KIX, bringing EYFP and ECFP close enough to observe the interaction by FRET. In perforated whole clamped cell, where the integrity of the cytoplasmic content is intact (Figure 7(A)), no activation of CREB transcription (Figure 7(B), panel (a)) and rearrangement of the *α*_1C,77_ C-tail (panel (b)) was observed when the C-tail was anchored to the plasma membrane. CREB transcriptional activity remained low in spite of a large sustained inward *I*_Ca_ (panel (d)) and the corresponding increase in [Ca^2+^]_i_ detected by the fluorescence of Ca^2+^ indicator Fura4 (panel (c)). Release of the *α*_1C,77_ C-tail by the activation of PIP_2_ hydrolysis upon activation of M1AchR (Figure 7(C)) at −90 mV caused significant elevation of [Ca^2+^]_i_ that also was not utilized by the cell for CREB transcriptional activation (Figure 7(C), panel (a)) until a depolarizing pulse to +20-mV was applied and the released *α*_1C,77_ C-tail was permitted to rearrange (Figure 7(D)). This experiment provides compelling evidence that neither large inward *I*_Ca_ nor the subsequent rise in [Ca^2+^]_i_ lead to CREB transcription activation, unless the conformational rearrangement of the *α*_1C,77_ subunit C-tail provides the precise targeting of the Ca^2+^ signal transduction (Figure 7(D), scheme) [66].

There is general agreement that CaM binds to LA and IQ domains of the *α*_1C_ C-tail, and acts as a sensor that conveys CDI (for review, see [63]). The affinity of CaM for both domains depends on [Ca^2+^]_i_. Our data indicate that CDI and Ca^2+^-signal transduction depend on the voltage-gated mobility of the *α*_1C,77_ C-tail. It is therefore reasonable to suggest that the LA-domain is a Ca^2+^-sensitive apo-CaM-mediated lock for the mechanism of slow voltage-dependent inactivation of the channel [67]. Apo-CaM associated with LA is able to cross-link it to another, still unidentified apo-CaM binding site in the polypeptide bundle underlying the pore. As the result of this specific localization, apo-CaM/LA “lock” is hidden from the cytoplasmic Ca^2+^ so that, for example, the intracellular Ca^2+^ released from the intracellular stores or Ca^2+^ caging compounds does not accelerate significantly the inactivation of Ca_v_1.2 [71]. Thus, apo-CaM associated with LA binds predominantly Ca^2+^ ions permeating through the pore. A Ca^2+^-dependent transfer of CaM from LA to the IQ-motif opens the “lock” and initiates a large rearrangement of the C-terminal tail. This in turn facilitates inactivation of the channel. The Ca^2+^/CaM complex with the IQ-motif is then transferred by the mobile C-tail to a downstream target of the Ca^2+^-signaling cascade (such as CaMKII [72]), where Ca^2+^ is released as an activating stimulus, while CaM switches back to LA and returns the C-tail to the resting position available for the next cycle of Ca^2+^-signal transduction.

**Figure 8:**
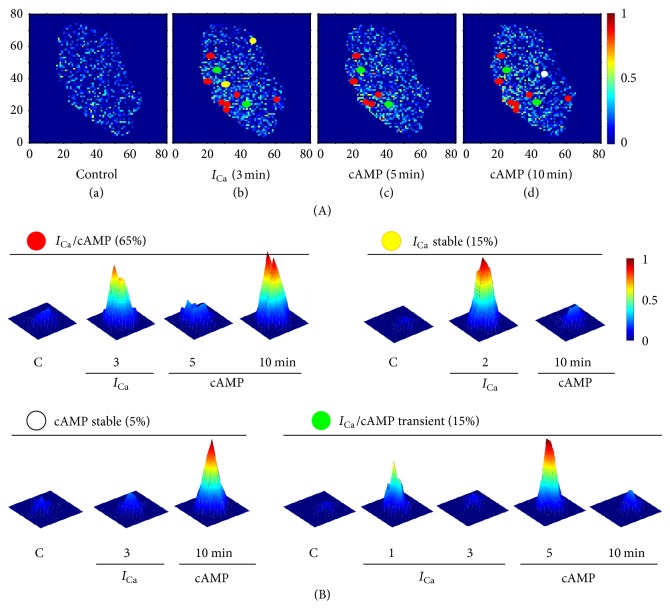
Microdomain analysis on the additive effect of *I*_Ca_ and cAMP on CREB signaling in COS1 cells expressing the recombinant “atherosclerotic” *α*_1C,77_ channel. (A) FRET signal within the nucleus during selected time points during *I*_Ca_ stimulation and cAMP application. Outlined are four types of signaling microdomains identified using 2D Mexican hat wavelet. Red circles represent stable microdomains that persist through both *I*_Ca_ and cAMP application. White and yellow circles show microdomains stable activated by cAMP and *I*_Ca_, respectively. Green circles represent transient microdomains of CREB signaling activation. Axes show pixel numbers. (B) Typical appearances of the four types of CREB signaling microdomains activated by *I*_Ca_ and/or cAMP application and recorded in their maximal development in relation to the time of applied stimuli. C: control recorded before stimulation. Color bars in (A) and (B) represent FRET values normalized to the maximum.

Activation of CREB-dependent transcription by the L-type *I*_Ca_ is mediated through multiple cell signaling pathways. Using FRET probes of CREB activity and 2D wavelet transform analysis, we applied principles of quantitative biology to detail the mechanism of Ca^2+^-activated CREB-dependent transcription within localized regions (microdomains) of the nucleus. We reached this goal by applying continuous wavelet analysis in two dimensions with a 2D wavelet as a deconvolution algorithm for FRET microscopy image analysis [73]. Continuous wavelet analysis is a mathematical technique that allows us to analyze a signal over several different frequencies across the entire signal [73, 74]. It is especially useful for finding heterogeneity in a signal because it can easily find where the pattern (i.e., frequency) of a signal changes. In these experiments, we, for the first time, obtained evidence of CREB signaling microdomains within the nucleus that respond differentially to *I*_Ca_ stimulation and cAMP (Figure 8(A)). Results of the study revealed that CREB-dependent transcriptional signaling occurs in discrete signaling microdomains underlying the architecture of nuclear signaling. Continuous activation of CREB-dependent transcriptional signaling by cAMP and Ca^2+^ resulted in a gradual increase of the number of microdomains. Four different categories of cAMP and Ca^2+^-induced CREB signaling microdomains were characterized in COS1 cells expressing recombinant Ca_v_1.2 with the “atherosclerotic” *α*_1C,77_ splice variant (Figure 8). In up to 65% of the microdomains, transcription was activated in additive manner by cAMP and Ca^2+^. Approximately 15% of signaling domains were activated only by *I*_Ca_ and 5% of domains were activated only by cAMP. Finally, 15% of the domains were transient, and activated by both cAMP and *I*_Ca_ (Figure 8(B)) [75]. A similar spatiotemporal organization of CREB-dependent signaling was observed in spontaneously beating neonatal rat cardiomyocytes. Although COS1 cells that were used in our experiments shown in Figures 7 and 8 are naturally deprived of Ca_v_1.2 [76], they inherited the ability to replicate the Ca_v_1.2-dependent activation of CREB signaling with the exogenous recombinant channel. Thus, this experimental approach fits the task, which, in my opinion, is the most important unresolved issue for the coupling of Ca_v_1.2 to CREB signaling: does the splice variation of *α*_1C_ affect the spatiotemporal organization of CREB-dependent signaling in a way that may affect cell proliferation and other crucial function?

## 7. Conclusions

Association of Ca_v_1.2 with regulation of transcription, cell proliferation, and its pathophysiology, as in the case of atherosclerosis, requires detailed investigation of the roles of the naturally occurring *α*_1C_ splice variants. It will limit the traditionally intuitive approach to Ca_v_1.2 in physiology and help to define new principle approaches to the treatment of various Ca_v_1.2 channelopathy-related dysfunctions, above all cardiovascular diseases. Humgenex Inc. provides consulting and logistic support on a broad range of issues reported in this review.

## Abbreviations

CaM: Calmodulin
CBD: Calmodulin-binding domain
CDI: Ca^2+^-dependent inactivation
DHP: Dihydropyridine
ECFP: Enhanced cyan fluorescent protein
EYFP: Enhanced yellow fluorescent protein
FALI: Fluorophore-assisted light inactivation
FRET: Fluorescent resonance energy transfer
[^3^H]PMD: 2,6-Dimethyl-3-methoxycarbonyl-5-([2,3-^3^H_2_]-n-propoxycarbonyl)-4-(2′-difluoromethoxyphenyl)-1,4-dihydropyridine
*I*_Ca_: Calcium current
PH: Pleckstrin homology domain
VSM: Vascular smooth muscle.
